# LATENT PROFILE ANALYSIS OF ANXIETY, DEPRESSION, ANGER, AND ADHD IN OLDER ADULTS

**DOI:** 10.1093/geroni/igz038.590

**Published:** 2019-11-08

**Authors:** Sarah M Israel, Erica Szkody, Michael R Nadorff, Daniel L Segal

**Affiliations:** 1 Mississippi State University, Mississippi State, Mississippi, United States; 2 University of Colorado at Colorado Springs, Colorado Springs, Colorado, United States

## Abstract

Older adults are generally happier, less likely to have depression or anxiety, and have better emotion regulation abilities than earlier in life. While older age predicts more hostile beliefs about others, older adults report less hostile behavior and no difference in covert hostility, compared to other age groups. However, brain regions associated with executive function and emotion regulation are impacted by even normal aging. Using latent profile analysis (LPA) we aimed to better understand what factors contribute to a dysregulated profile in older adults and how age altered the dysregulation profile. The current archival study includes data from 518 older adults between the ages of 60 and 95 years (M = 70.73, SD = 7.34). Participants completed the Coolidge Axis II Inventory (CATI) database. The CATI is a 250-item psychopathology and neuropsychological inventory that assesses over 40 clinical and neuropsychological disorders utilizing official DSM-5 criteria. A Dysregulated Profile was identified using an LPA of diagnosis subscales (i.e., Anxiety, Depression, Anger, and ADHD) that have been previously associated with dysregulation in children and young adults. Results demonstrated that female participants reported more ADHD symptoms (more impairment in executive function) than men. Furthermore, the dysregulated profile (high on all subscales) and age interacted such that, as age increased, scores on the Depression and Anger subscales decreased. No significant differences were found for any other interactions. Our findings are consistent with existing literature. Even in the dysregulated profile, participants reported less anger and depression with older age.

